# Mannitol as adjunct therapy for childhood cerebral malaria in Uganda: A randomized clinical trial

**DOI:** 10.1186/1475-2875-6-138

**Published:** 2007-10-24

**Authors:** Beatrice Namutangula, Grace Ndeezi, Justus S Byarugaba, James K Tumwine

**Affiliations:** 1 Department of Paediatrics and Child Health, Makerere University Medical School, P O Box 7072 Kampala Uganda

## Abstract

**Background:**

Several reports have suggested that raised intracranial pressure (ICP) is a major contributor to death among children with cerebral malaria. Mannitol, an osmotic diuretic, effectively lowers ICP and is used to treat post-traumatic raised ICP. It is not clear whether intravenous mannitol given to children with cerebral malaria improves clinical outcome. The objective of this study was to determine the effect of mannitol as adjunct therapy on the clinical outcome of children with cerebral malaria.

**Methods:**

This randomized double-blind placebo controlled clinical trial was carried out at the Emergency Paediatric ward of Mulago Hospital, Uganda's national referral and teaching hospital. One hundred and fifty six children aged 6 to 60 months with cerebral malaria were randomized to either one dose of mannitol 1 g/kg or placebo, in addition to intravenous quinine. Main outcome measures included coma recovery time; time to sit unsupported, begin oral intake; duration of hospitalization; death and adverse effects.

**Results:**

Time to regain consciousness (p = 0.11), sit unsupported (p = 0.81), time to start oral intake (p = 0.13) and total coma duration (p = 0.07) were similar in both groups. There was no significant difference in the mortality between the placebo (13/80 or 16.3%) and mannitol (10/76 or 13.2%) groups: RR = 1.2 (CI 0.5–2.7). No adverse effects were observed after administration of mannitol.

**Conclusion:**

Mannitol had no significant impact on clinical outcome of cerebral malaria. It is difficult to recommend intravenous mannitol as adjunct therapy for childhood cerebral malaria.

**Clinical registration number:**

ClinicalTrials.gov ID: NCT00113854

## Background

Cerebral malaria is one of the most severe and life threatening complications of *Plasmodium falciparum *infection accounting for high mortality [1-3]. Several studies have shown evidence of raised intracranial pressure in children with cerebral malaria and this may be associated with poor outcome[4,5].

Mannitol, an osmotic diuretic lowers intracranial pressure by creating an osmotic gradient that draws water from brain parenchyma into the brain capillaries. It also slows production of cerebral spinal fluid, which further reduces intracranial pressure [6]. Although recommended and used to treat post-traumatic comatose patients[6,7] it is not yet recommended in cerebral malaria due to lack of conclusive evidence. No randomized or quasi-randomized controlled trials support or refute the use of mannitol as adjunct treatment for cerebral malaria. A randomized trial was performed to determine the effect of mannitol as adjunct therapy on the clinical outcome of cerebral malaria in children.

## Methods

Patients were enrolled from October 2004 to May 2005 in the Paediatric Emergency ward of Mulago Hospital, Uganda's national referral and teaching hospital.

### Selection criteria

Children aged between six months and five years, who fulfilled the WHO case definition of cerebral malaria (unrousable coma lasting more than 30 minutes after a seizure with peripheral asexual *P. falciparum *parasitaemia and absence of other causes of coma) and those whose caretakers gave informed consent were included. Those children who had been sedated within two hours before admission; those with overt signs of pulmonary congestion, heart failure, a history of renal disease or had not passed urine within 24 hours before admission were excluded.

### Randomization and blinding

The caretakers and investigators were blinded to group assignments. Study numbers were computer generated and sent to the manufacturer for labeling of the drug bottles. Randomization was done in blocks of variable sizes (4–10) to ensure equal distribution in each group. Patients were randomized to receive either mannitol or placebo in addition to treatment for severe malaria (intravenous quinine). Both mannitol and placebo were administered as intravenous infusions over 20 minutes. One of the investigators (BN) and an assistant, both of whom were blinded to the treatment, carried out observations.

### Treatment

All patients received intravenous quinine dihydrochloride 10 mg salt/kg body weight in 10 mls/kg of 5% dextrose, which was infused over 2–4 hours and repeated every eight hours till the patient regained consciousness. Quinine sulphate was then given orally (10 mg salt/kg) every eight hours till a total of seven days was reached.

Patients in the mannitol group received 5 ml/kg of 20% (1 g/kg) intravenous mannitol over 20 minutes, while those in the placebo group received 5 ml/kg of normal saline over 20 min in addition to intravenous quinine. The placebo bottles contained 100 ml of normal saline while the mannitol bottles contained 100 ml of 20% mannitol. Supportive measures included control of seizures, hypoglycaemia, severe anaemia and fever.

Feeding was through a naso-gastric tube until the child was able to take orally. Patients were followed daily until discharge, death or up to day 7. The level of consciousness was assessed three hourly using the Blantyre coma scale until full consciousness was regained.

### Laboratory tests

These included cerebral spinal fluid analysis, complete blood count, liver function tests and blood sugar (day 0), renal function tests (day 0 repeated on day 3), and peripheral blood smear for malaria parasites (day 0, day 3 and day 7). Malaria parasite density was determined by Giemsa-stained thick films and counted per 200 white blood cells; the results were expressed as parasites per μL assuming a total white blood cell count of 8,000× 106/L.

### Main outcome measures

These included: time to regain consciousness (from the start of treatment till the Blantyre coma scale reached 5); time to sit unsupported ;time to start oral feeds; and duration of hospital stay (from start of treatment to discharge).

### Sample size and statistical issues

A sample size of 78 patients in each group for 90% power and 95% confidence interval was calculated, with an assumption that 41% of the children would have regained consciousness by the first 24 hours[8]. An assumption was also made that this would increase to 66% with mannitol. Analysis was by intention to treat. Differences in the two groups were compared using student t tests, the Mann Whitney U test (where appropriate); the log rank tests for continuous outcomes, and the chi squared test and odds ratios and risk ratios for categorical variables.

### Ethical issues

Permission to conduct the study was obtained from the Uganda National Council for Science and Technology, Makerere University Medical School and Mulago hospital Research and Ethics committees. Parents gave informed consent for the children's participation in the study.

## Results

**Figure 1:**
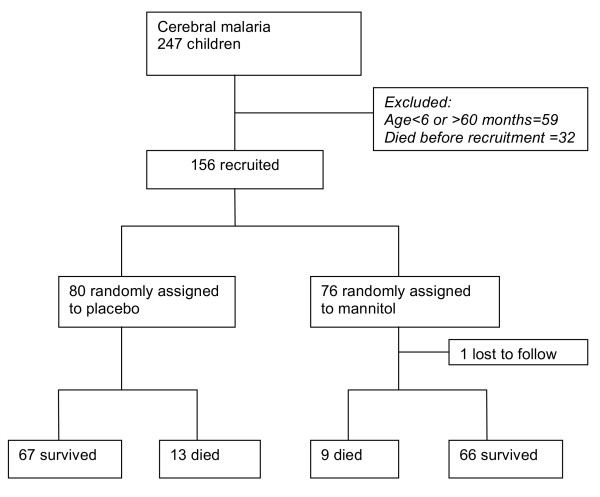
Trial profile.

Two hundred and forty seven children were screened for eligibility and 156 were enrolled and treated. Seventy-six patients were randomly assigned to receive mannitol while 80 patients received placebo (Figure 1). Baseline characteristics were comparable in both groups (Table 1). For example the commonest symptoms were fever (98.7%) and convulsion (98.7%). The mean number of convulsions on admission was 3.8 (SD 2.8) in the placebo group and 4.1 (SD 2.5) in the mannitol group

**Table 1:** Baseline clinical characteristic of the 156 patients with cerebral malaria in both treatment arms on admission.

**Variable**	**Placebo** N = 80	**Mannitol** N = 76	**P value**
Female	42(52.5%)	34(44.7%)	0.33
Fever	79(98.8)	76 (100%)	0.33
Convulsions	79(98.8%)	75(98.7%)	0.97
Duration of coma	7.0 (IQR3.5–12.0)	6.0 (5.0–12.0)	0.79
Blantyre coma score 1/5	13(16.2%)	10(13.2%)	0.59
Abnormal fundoscopic changes^a^	28(35.0%))	16(21.1%)	0.53
Papilloedema	3(3.8%)	5(6.6%)	0.42
Sodium – mmol/l (SD)	133.8 (4.2)	130.9 (14.8)	0.09
Hb – g/dl (SD)	7.1 (1.8)	7.1 (1.9)	0.98
Parasite density/μL (IQR)	37000(5390–115760)	58860(12390–284590)	0.05

IQR Inter Quartile Range

^a^Abnormal fundoscopic changes (congested retinal veins, blurred disc margins, papilloema, retinal haemorrhages) indicative of raised intracranial pressure

**Figure 2:**
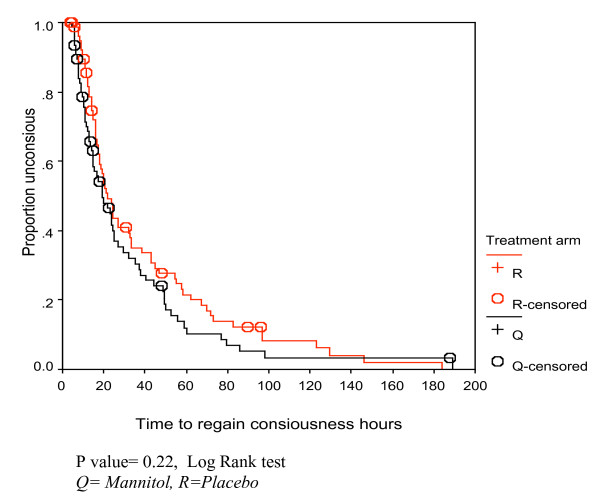
Kaplan Meir survival curve for time to regain consciousness (hours) by treatment group among the children with cerebral malaria.

**Figure 3:**
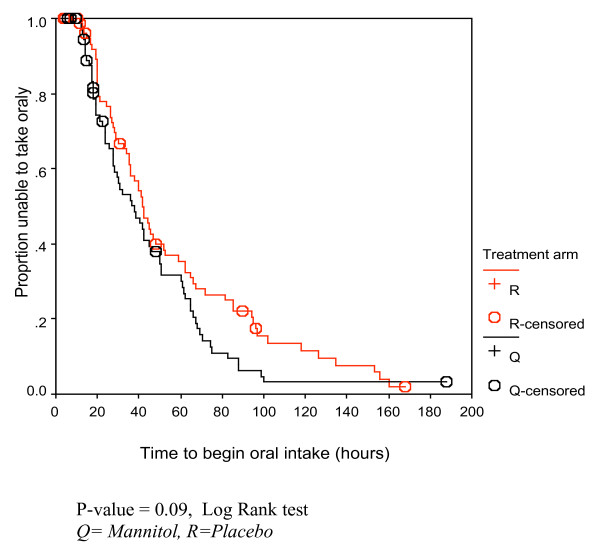
Kaplan Meier curve for time to begin oral intake (hours) for the children with cerebral malaria in the two treatment arms.

**Figure 4:**
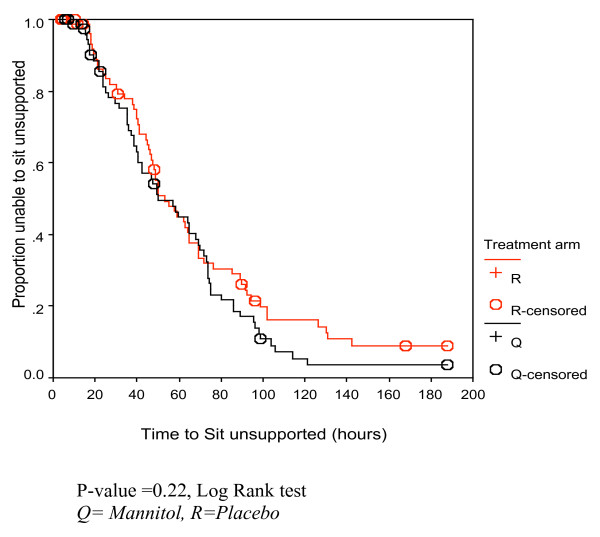
Kaplan Meier curve for time to sit unsupported (in hours) for the children in the two treatment arms.

Generally there was no significant difference in the clinical outcomes (Table 2) between the two treatment groups. Kaplan Meier survival analysis revealed similar trends in the two treatment groups, with the log rank tests showing no significant difference (Figures 2, 3, 4).

**Table 2:** Outcome of 133 children who survived with cerebral malaria in the two groups

Outcome	Placebo(N = 67) Median (IQR)	Mannitol(N = 66) Median (IQR))	P value*
Time [hours] to regain consciousness	20.5(14.1–53.4)	18.9(10.0–38.0)	0.11
Time [hours] to oral intake	40.0(23.3–66.3)	34.0(19.0–61.0)	0.13
Time [hours] to sit unsupported	49.0(33.0–69.8)	49.0(29.0–74.0)	0.81
Total coma duration	46.3(33.5–81.4)	43.0(22.9–70.0)	0.07

CI = Confidence Interval

* Mann-Whitney U-test

### Adverse effects

There was no hypersensitivity reaction or vomiting noted in any of the children after receiving mannitol. There was no significant change in the renal function, pulse, or blood pressure.

Twenty two children died and one was lost to follow up. Since the analysis was by intention to treat, this patient was assigned the worst possible outcome (death). This brought the number of deaths to 23 (14.7% overall mortality); that is 10 (13.2%: CI 7.1–22.8%) in the mannitol group and 13 (16.3%: CI 9.6–26.0%) in the placebo group. The difference in mortlity between the two groups was not statistically significant (RR = 1.2 (CI 0.5–2.7). Post-mortem examination was done on 6 children whose caretakers consented. Five children had signs of anoxia and cerebral oedema while the sixth child had signs of acute tubular necrosis.

## Discussion

The aim of this study was to determine the effect of mannitol as adjunct therapy on the clinical outcome of children with cerebral malaria. The differences in clinical outcomes between the mannitol and placebo groups were generally not significant.

### Time to regain consciousness

Median time to regain consciousness was shorter in the mannitol group (18.9 hours) than in the placebo group (20.5 hours), but this was not statistically significant. A few studies have assessed time to regain consciousness using other adjunct therapies such as iron chelation [9] and dexamethasone [10]. When intravenous mannitol is compared to these interventions [9,10], the time to regain consciousness is much shorter. In the current study, the rate of recovery from coma among patients receiving mannitol was 1.6 times that of patients on placebo.

Failure to demonstrate a significant effect may be due to the fact that coma in cerebral malaria is a result of multiple factors of which cerebral oedema [11] and increased intracranial pressure [12] are just a part. Unfortunately, intracranial pressure was not measured, so it is not possible to confirm whether it was a major contributor to coma. Although the diagnosis of malaria is straight forward, not all patients fulfilling the WHO definition of cerebral malaria have the disease especially in areas where *P. falciparum *parasitaemia is often asymptomatic. It is possible that imprecise diagnosis may mask the benefits of an intervention such as mannitol.

It is possible that the dose (1 g/kg) of mannitol used was small hence the lack of a significant difference in time to regain consciousness. Mannitol doses ranging from 0.25 g/kg to 2.5 g/kg have been used in some studies [13,14]. Bolus doses of 0.25/kg, 0.5 g/kg, > 1.0 g/kg reduce intracranial pressure in patients with head injury by 25%, 78% and 98% respectively [13]. In Kenya, Newton *et al *[5] gave 0.5–1 g/kg of intravenous mannitol to children with cerebral malaria, while in Ghana, mannitol was given at a higher dose (1 g/kg) 8 hourly[15]. Both studies, showed a positive effect of mannitol on coma recovery for children who received it. However, these studies were not designed to assess the effect of mannitol as adjunct therapy in cerebral malaria.

### Time to begin oral intake

The median time taken to begin oral intake was shorter in the mannitol group (34.0 hours) than in the placebo group (40.0 hours), but the difference was not statistically significant (Mann Whitney-U Test).

### Time to sit unsupported

There was no significant difference between the two groups in the time taken to sit unsupported. The children in the mannitol group regained consciousness earlier than those in the placebo group hence were expected to sit unsupported earlier than those in the placebo group.

### Mortality

The difference in mortality between the two groups was not significant but was comparable to ranges of 7–17% reported in other Uganda[3,16] and African[1,9,17] studies.

Although this study was not powered to detect differences in mortality, the fact that fewer children died in the mannitol group compared to the placebo group and that the mortality was within the ranges seen in most African studies, may be an indication that mannitol is not deleterious. However this needs to be tested with a larger sample size.

Only six of the 23 children who died had post-mortem examinations done as consent was declined in the remaining children. All but one of these six children had succumbed to cerebral malaria as evidenced by cerebral vascular congestion, petechial hemorrhages in the brain and a histological picture consistent with malarial trophozoite invasion of the RBCs in the cerebral vasculature. These findings are similar to those found in other studies [3,8]. One child had evidence of acute renal failure and it is very likely that the acute tubular necrosis, which resulted in his death, was a result of malaria. This child was from the placebo group, hence mannitol could not have caused his renal impairment. Renal impairment is common in severe malaria[18] and though it rarely progresses into frank renal failure, it is associated with poor outcome. Factors, which significantly predicted mortality, included; multiple convulsions (> 5), deep coma (BCS of <2/5), leucocytosis and abnormal fundoscopic findings (papilloedema, retinal haemorrhages and retinal vein congestion), which are similar to those, described in earlier studies [1,19,20].

### Adverse effects

Apart from death, no major adverse effects were identified. Deaths in the mannitol group were probably due to the cerebral malaria and not the mannitol since the mortality was within the ranges of other studies and was comparable with the number of deaths in the placebo group. Mannitol (1 g/kg) did not significantly affect renal function in the children with cerebral malaria. There was also no significant difference in the blood pressure and pulse changes between the two groups. Other adverse effects such as exacerbation of heart failure and pulmonary oedema were not observed.

## Conclusion

In conclusion, mannitol did not significantly reduce time taken to regain consciousness, sit unsupported, or mortality. It is difficult to recommend intravenous mannitol as adjunct therapy for childhood cerebral malaria.

## Authors' contributions

BN wrote the proposal, recruited and followed up patients and wrote the manuscript. GN revised the proposal, supervised the study and revised the manuscript. JB read through the proposal and manuscript and supervised the study. JKT revised the proposal, supervised the study, analyzed the data and wrote the manuscript.
